# The Role of a Ministry of Education in Addressing Distance Education during Emergency Education

**DOI:** 10.3390/ejihpe12050036

**Published:** 2022-05-20

**Authors:** Wajeeh Daher, Huda Salameh

**Affiliations:** 1 Department of Educational Administration, Arab American University, Ramallah P600, Palestine; huda.ahmad@moe.edu.ps; 2 Department of Educational Sciences, An-Najah National University, Nablus P400, Palestine; 3 Ministry of Education, Ramallah P600, Palestine

**Keywords:** distance education, education, ministry of education, emergency education, crisis education

## Abstract

The present study aims to identify the role of a Ministry of Education in meeting the challenges faced due to distance education as emergency education. The study participants were nine officials working at the Palestinian Ministry of Education and Higher Education. We used interviews to collect data and inductive content analysis to analyze these data. The study result indicates that the ministry carried out action related to the different educational aspects to meet distance education challenges. It is recommended that ministries of education strengthen their collaboration with the local community. The aim of this collaboration is two-fold: encouraging parents’ support of technology integration in education and encouraging their role in positively influencing their students’ perceptions of the use of ICT in learning. Teachers also need to engage in the change of students’ perception towards a more positive one regarding the influence of ICT on learning.

## 1. Introduction

Research on distance education in the pandemic is flourishing as the world experiences COVID-19 and its educational consequences (e.g., [1,2]). Tomczyk and Walker [3] argue that generally, emergency e-learning due to the COVID-19 pandemic took many by surprise, but in particular it was hard in communities and groups with fewer resources and weak Internet infrastructure. Moreover, the outbreak of the COVID-19 pandemic revealed weaknesses that existed in pre-pandemic education systems and added new challenges [4]. Specifically, this outbreak requested immediate actions from ministries of education all over the world. Though some research has appeared regarding the roles of ministries of education to confront the COVID-19 pandemic educational results (ex., [5] on Latin America), little research has been carried out on the steps taken by the ministries of education in developing countries such as Palestine to address distance education during the COVID-19 pandemic. To attempt to bridge this lack of research, the present research interviewed officials from the Palestinian Ministry of Education and analyzed texts that the Ministry published on its website, or other websites as those of news agencies. The officials were asked about the actions taken by the Ministry of Education to confront the challenges of distance learning education in a time of emergency.

### 1.1. Literature Review

The literature review will target topics related to the present research: distance learning in schools; emergency education; policies carried out by different countries during the COVID-19 pandemic; Information and Communication Technology (ICT)-based education in Palestine and dimensions of quality in education administration.

#### 1.1.1. Distance Learning in the Schools

Fidalgo et al. [6] say that the World Wide Web helped a large fraction of the world’s population have access to information, which supported the distribution of educational content, and thus assisted in moving distance education to the digital era. Researchers who came to define and describe distance education emphasized that this educational experience occurs in a context where instructors and learners are separated in time and space [7]. There is no requirement that you attend an academic institution to achieve a degree or credential [8].

Distance, online and blended learning impacts positively students’ learning, including their learning outcomes [9]), their affective learning [10], the metacognitive potential of learning [11], their motivation [12], their interaction [13] and their identity [14]. However, this positive impact of distance education may not always be valid. Parkinson et al. [15] found that distance education has benefits for off-campus students by providing easier access to educational opportunities, but they also found that in a traditional classroom setting, on-campus students have greater satisfaction with their learning experiences.

#### 1.1.2. Emergency Education

Researchers have been interested in emergency education since the COVID-19 outbreak (ex., [16,17,18]). Sofianidis et al. [4] argue that the COVID-19 pandemic created unique opportunities for the digital transformation of education, but at the same time, it has highlighted various shortcomings of the current educational system. They further argue that we need to overcome the challenges of digital education to effectively utilize the educational potential of digital technologies, especially distance, online and blended learning.

According to data from the Economic Commission for Latin America and the Caribbean—United Nations Educational, Scientific and Cultural Organization (ECLAC-UNESCO), by mid-May 2020, more than 1.2 billion students at all levels of education worldwide had stopped having face-to-face classes [5]. ECLAC-UNESCO [5] reported that even before the pandemic came, the social situation in the region was deteriorating due to rising rates of poverty, the persistence of inequalities and growing social discontent. This economic situation had a negative impact on the various social sectors, particularly education. The previous claim applies to Palestine, with its prevailing digital divide [19]. According to Schleicher [20], this digital divide is worsened in emergency education, since privileged students are able to make their way through closed school doors to alternative learning opportunities, while disadvantaged students are often shut out.

Researchers studied the educational experiences in Palestine during emergency education. Shraim and Crompton [21] found that Palestinian teachers and decision-makers identified mobile devices, social media and cloud computing as tools for designing and delivering educational materials, as well as for communicating effectively during the COVID-19 epidemic. Moreover, Shraim and Crompton identified different challenges, including the expanding of education’s digital divide and a negative attitude towards online learning.

#### 1.1.3. Policies for Successful Education during the COVID-19 Pandemic

ECLAC-UNESCO [5] reported that actions taken by ministries of education of countries in Latin America and the Caribbean concerned different aspects of online learning, including the preparation of teachers for online learning. For example, Ecuador’s Ministry of Education launched a teachers’ course named “My Online Classroom” that was based on self-learning. Another aspect is the establishment of digital devices for teachers and students, as part of the digitalizing education. This establishment was sometimes achieved through loans to teachers. Moreover, ECLAC-UNESCO [5] says that countries established ways through various distance learning modes.

To study the efficiency of the policy followed by the Ministry of Education, Science and Technology in Nepal, Shrestha and Gnawali [22] analyzed the educational policy documents issued by the Ministry. The analysis revealed several strengths of the policy, such as planning to create data in terms of learners’ access to resources, encouraging learners to value self-learning and parent education, and suggesting several alternative ways to resume school. Shrestha and Gnawali [22] recommended that in any future policy, teachers should have the autonomy to decide the course content in addition to creating the course content. The previous autonomy would enable teachers to comfortably and realistically meet the learning objectives of the curriculum and acknowledge their worthiness as teachers [22].

#### 1.1.4. ICT-Based Education in Palestine

ICT-based education in Palestine was initiated and maintained by the involvement of the Palestinian Ministry of Education and Higher Education, by the Palestinian local support, and by European-funded projects. Below, we elaborate on each of the local-support and European-funded initiatives and projects.

Wahbeh [23] studied ICT implementation in Palestinian schools. The author describes the relationship, related to ICT in schools, between the Ministry of Education and Higher Education (MoEHE), the local community or the parents’ associations (PTAs), and the school administration. The description shows that the technical infrastructure for the internet was provided by MoEHE and the PTA, with the help of the local community. It also reveals that, as a result of the Ministry of Education’s bureaucratic measures, the schools were slowed in connecting to the internet.

Having explored how teachers use computer technology in schools and how the Palestinian Ministry of Education and Higher Education views computer integration into schools, Barham [24] concluded that the impact that computer technology can have depends on the design of the teaching and learning environment supporting student-centered learning. She recommended that the Palestinian Ministry of Education and Higher Education should work to develop classroom environments that integrate technology into the learning process. She stressed that the availability of internal resources will determine whether the Ministry can achieve this goal. Furthermore, she argued that the Ministry may need outside donations to accomplish its mission due to the lack of resources in the country. Thus, Barham [24] emphasizes two issues. Firstly, there were challenges with the integration of ICT in the Palestinian classroom, and secondly, in order to overcome these challenges, it was necessary to develop internal resources.

European-funded projects were carried out in Palestine with the help of the Palestinian Ministry of Education and Higher Education to improve digital literacy and encourage the use of ICT in schools. We describe two of these projects here. The first project was funded by an Italian Cooperation, managed by the United Nations Development Program, and involved the Palestinian Ministry of Education and Higher Education. Pacetti [25] described the project as encouraging ICT use in Palestinian schools and universities. From the report of Pacetti, we see that universities took a main role in preparing appropriate materials, models of use of technology, and strategies for this use. Specifically, the University of Bologna had a main role in the project, implementing the ICT knowledge and critical practice in the schools and providing pedagogical models of the use of ICT, tools, and methodologies, which helped monitoring the pedagogical experimentation of the use of ICT in the classroom.

The second project was funded by the Belgian Development Agency (ENABLE) and involved the Palestinian Ministry of Education and Higher Education. ENABLE [26] described the previous project as follows: “288 pilot schools participated and were rewarded with ICT material based on their own ICT-needs analysis. By the end of the project, a total of 1600 learning objects were developed by teachers in a successful bottom-up approach and uploaded to the teacher web portal developed by the Ministry of Education”. Referring to the previous project, Traxler [27] says that in 2016, a set of Policy Papers were developed in the context of an E-learning project, jointly undertaken by the Palestinian Ministry of Higher Education and the Belgian Development Cooperation. The project aimed to introduce the use of ICT in the school curriculum to encourage the role of the student in the classroom, as well as acquiring 21st Century Skills in Palestine. Three policy papers focused on school processes and the fourth focused on digital literacy. Traxler [27] argues that “The status of the Papers is unclear—they are certainly not policy—but these paragraphs represent a significant milestone” (p. 7).

#### 1.1.5. Dimensions of Quality in the Administration of Education

Kivistö and Pekkola [28] utilized Harvey and Green’s [29] framework for quality and the conceptualization of administration to develop a framework for quality education. This framework is composed of five dimensions. The first dimension is called “Administrative quality as exceptionality/excellence”. In the first dimension, we have “tangible” factors such as attractiveness and adequacy of facilities, but we also need a budget appropriate for the level of resources and well-qualified motivated staff. Here, benchmarking against an acceptable minimum set of standards is one of the ways to determine quality, at least to some degree. The second dimension is called “Administrative quality as perfection/consistency”. This dimension is concerned that aspects of administrative work must be reliable, accessible, and accurate. Aside from the level of service provided externally and internally, perfection also includes a level of responsiveness or staff willingness to assist. The third dimension is “Administrative quality as fitness for purpose”. This dimension refers to meeting the expectations of internal and external users for any administrative service. This dimension also refers to an academic institution’s ability and capacity to perform its mission and goals by fulfilling the purpose of its administration (or some part of it). The fourth dimension is “Administrative quality as value for money”. Here, quality is considered as being able to maximize the benefit from administrative services given limited resources, including monetary and human resources. The fifth dimension is “Administrative quality as transformation”. The fifth dimension considers whether the administration provided support for academic activities at universities, but they also proactively create conditions for academic excellence, financial success, and student needs—both at the same time.

### 1.2. Significance of the Study

Previous research that studied education in Palestine during COVID-19 targeted teachers’ and students’ behavior and perceptions (e.g., [21,30]). The present study highlights the role of the Palestinian Ministry of Education in addressing the challenges faced by the schools in turning into distance education. This study thus addresses the processes undertaken by the Ministry of Education in a country that is considered a developing country [31], namely Palestine, during the Coronavirus pandemic that enforced emergency education represented by distance learning. Distance learning is new as a means for education in schools, which points to the importance of studying this phenomenon at the administrative level. This level has been paid little attention to, indicating the need to study it.

### 1.3. Research Question

What actions have been undertaken by the Palestinian Ministry of Education to address distance learning during the COVID-19 pandemic?

## 2. Materials and Methods

### 2.1. Research Setting and Participants

The participants in the present study were Ministry of Education officials who were involved with distance learning during the COVID-19 pandemic. Those participants were three officials from the supervision and qualification department (SQD), three supervisors from the General Administration of Educational Technologies and Information Technology (AETIT), and three training supervisors from the National Institute of Educational Training (NIET).

The sample is a convenient one. We approached 4–5 supervisors from each department at the Palestinian Ministry of Education who took responsibility for distance education during the COVID-19 pandemic: the SQD, the AETIT and the NIET. We interviewed those who accepted our request—three from each department. Table 1 describes the nine participants in terms of department and seniority at work. All names are fictive.

### 2.2. Data-Collecting Tools

A couple of tools were used: semi-structured interviews and news on the site of the Palestinian Ministry of Education. We detail each one of the collecting tools below.

#### 2.2.1. The Semi-Structured Interview

The semi-structured interview of the Ministry’s officials contained one question: what procedures did your department in the Ministry perform to meet the challenges of distance learning in the schools during the COVID-19 pandemic?

#### 2.2.2. Reviewing the Ministry’s News Published on Websites

We searched for the Ministry’s news published on websites, especially the website of the Ministry of Education. The three main terms used in the search were “ministry of education”, “Palestine”, and “COVID-19” or “Coronavirus”.

Formal consent form:

All the participants signed a formal consent form that included their agreement to participate in the research on the actions carried out by the Palestinian Ministry of Education during emergency education.

### 2.3. Data Analysis Tools

In the present research, we used inductive and deductive content analysis. In the inductive analysis, we tried to find out the categories as they emerged from the interviewees’ responses. In the deductive analysis, we depended on the administration’s quality framework of Kivistö and Pekkola [28], and its five dimensions: administrative quality as exceptionality/excellence, administrative quality as perfection/consistency, administrative quality as fitness for purpose, administrative quality as value for money and administrative quality as transformation. These categories helped us deepen our understanding of the emerging categories.

Validity and reliability of the analyzing process:

To evaluate the analysis method, we used the criteria described in Lincoln and Guba [32]. The first criterion is trustworthiness. Trustworthiness aims to establish the legitimacy of the inquiry’s findings so that they are worth attention [32]. Elo et al. [33] say that inductive content analysis is particularly important here, as it creates categories from raw data without reference to a theory-based categorization. Lincoln and Guba [32] have proposed four alternatives for assessing the trustworthiness of qualitative research: credibility, dependability, conformability and transferability. In 1994, the authors added authenticity as a fifth criterion. Below, we address each component as related to the present research. We do this depending on Elo et al. [33].

First component: Credibility and dependability:

Definition:

To establish credibility, the research participants should be identified and described accurately. This is also related to dependability, which refers to the stability of data over time and under different conditions. Elo et al. [33] say that this could be achieved when the principles and criteria used to select participants are clear, and when we detail the participants’ main characteristics so that the transferability of the results to other contexts can be assessed [34].

Application in the present research:

Here, we decided to interview officials from the three ministries of education departments that were responsible for addressing the distance learning issues: Administration of Educational Technologies and Information Technology, the NIET. Above, we described the participants in terms of age and seniority in the department. In addition, researchers say that credibility, in qualitative content analysis, is studied in terms of the homogeneity of the study participants or differences expected between them [35]. We took care in the present research of both sides. Homogeneity was satisfied when we interviewed more than one participant in the same department, while differences were taken care of by interviewing the different Ministry of Education departments that were directly involved with distance education.

Second component: Conformability:

Definition:

This refers to objectivity; that is, the potential for congruence between two or more independent people about the accuracy, relevance or meaning of the data. Conformability of data means that the findings accurately represent the information that the participants provided, and the interpretations of those data are not invented by the inquirer [36]. Researchers emphasize comfortability when analyzing latent content as gestures, in addition to manifest content [37] as it may result in overinterpretation [33].

Application in the present research:

In the present research, we addressed conformability in two ways. First, by triangulation, as we collected data by two methods, intending to assess the accuracy of our finding based on one collecting method against that based on the other collecting method. In addition, we computed the agreement between coders. Two experienced coders (the two authors) coded the resulting themes and categories, searching for occurrences of sentences that indicated a Ministry of Education’s activities related to distance learning in schools during the COVID-19 pandemic. The agreement between the coders, computed here through Cohen’s Kappa coefficient, indicates the reliability of the qualitative coding, when satisfied. This computation, for the various categories related to the Ministry of Education actions, resulted in values between 0.85 and 0.91. The previous values are considered acceptable for reliability according to the agreement between coders.

Third component: Transferability

Definition:

This refers to the potential for extrapolation. It relies on the reasoning that findings can be generalized or transferred to other groups of participants or settings. Transferability increases in giving clear descriptions of the context, selection and characteristics of participants. Trustworthiness is increased if the results are presented in a way that allows the reader to look for alternative interpretations [38].

Application in the present research:

We tried to take care of transferability by detailing the research setting above, as well as the description of the participants. It is also possible to ensure transferability by showing how the collected data were analyzed. This is described in Table 2 below.

Fourth component: Authenticity

Description:

This refers to the extent to which researchers, fairly and faithfully, show a range of realities ([32,36]). Authenticity could suffer from inaccurate analysis of inexperienced researchers who do not have the knowledge and skills required. This could happen when the researcher is unable to use and report the results correctly.

Application in the present research:

Our use of thematic analysis and constant comparison method lessens the possibility of inaccurate analysis, in addition to our computation of the agreement between judges. In presenting the results, we follow different qualitative studies ([39,40]).

## 3. Results

The present research came to answer the question: what actions have been taken by the Palestinian Ministry of Education to address distance learning during emergency education? To answer the question, a group of staff members of the Ministry of Education was interviewed about the different aspects of distance learning that they attended during emergency education. The following categories emerged from the interviews. For each category, we detail the themes emerging in this category.

### 3.1. Students’ Preparedness and Attendance

The category “Students’ preparedness and attendance” had three sub-categories, as described below.

#### 3.1.1. Concern with Students’ Preparedness for Distance Learning

Collected data suggest that the Ministry of Education was concerned with students’ preparedness for distance learning. P1, from SQD, said:

*Students have been trained how to manage Teams, whether alone or with their parents. They were trained to perform tasks, whether forum tasks or worksheets, or tests. They learned how to send the tasks to the teacher to receive feedback*.

The previous sub-category is related to the quality dimension “administrative quality as fitness for purpose”, where the purpose of the Ministry of Education was to help students be prepared for distance learning.

#### 3.1.2. Concern with K2 Students

P1 asserted that the Ministry of Education was especially concerned regarding the learning of K2. She said:

*The ministry was especially concerned with first and second-grade students. K2 schools were requested to educate children, together with their parents how to work with Teams. The ministry followed up whether parents had participated in the workshops.*

The previous sub-category is also related to the quality dimension “administrative quality as fitness for purpose”. Here, the purpose was the preparedness of a specific student population, namely K2 students.

#### 3.1.3. Concern for Students’ Preparedness for Special Occasions

This concern with students’ preparedness for online teaching and learning was also present on special occasions as the Program for International Student Assessment (PISA). P2, from SQD, said:

*The ministry of education was concerned with preparing the students for participating in the Program for International Student Assessment (PISA). To do so it uploaded to its site materials relevant for the assessment. These materials contained text files as well as video files. The goal of the video files was to help students with the assessment in times of emergency.*

The previous sub-category is also related to the quality dimension “administrative quality as fitness for purpose”. Here, the Ministry of Education was concerned with the preparedness of students in special occasions. This concern is understood as special occasions could be different than regular occasions, which points at the need to take special care of them.

### 3.2. Teacher’s Skills and Qualifications

The category “Teacher’s skills and qualification” had two sub-categories, as described below.

#### 3.2.1. Qualifying Teachers for Distance Teaching

The participants emphasized the Ministry of Education’s effort to qualify teachers for distance teaching. P9, from the AETIT, said: “The teachers were requested to participate in workshops that prepared them to work with platforms like Teams, and to use technological tools in their teaching”.

The previous sub-category is also related to the “administrative quality as fitness for purpose” dimension, and is also related to administrative quality as perfection/consistency. Participating in workshops could involve fruitful discussion that results in consistency in teaching methods, as well as the perfection of this teaching method.

P5, from the NIET, said:

*The National Institute of the ministry of education runs a teacher qualification program called the diploma of professional qualification of the expert teacher. This program contains six competencies established based on the ministry’s specific educational needs at that year. This year, after the COVID-19 pandemic, the program has targeted pedagogical and technological pedagogical content knowledge (TPACK). It included interactive sharing, electronic activities, electronic evaluation, continuous communication, employing the Palestinian channel effectively. This program emphasized teaching competencies, learning patterns, and teaching and learning strategies.*

P5′s description is related to qualifying teachers for distance education as related to the category “administrative quality as perfection/consistency”. The skills learnt in the program are expected to move the participating teachers into advancing their distance teaching towards more perfect one.

#### 3.2.2. Developing Teachers’ Skills in the Use of Technological Tools

The participants emphasized the Ministry of Education’s effort to qualify teachers for effective use of technological tools. P4, from the NIET, noted: “We focused on developing teacher skills in the use of technological tools. Specifically, we developed printing mathematics symbols (math lap), an office 365 package, electronic test models on Google, in addition to converting the PowerPoint presentations into videos.”

P4′s description of developing teachers’ skills in the use of technological tools is related to the category “administrative quality as perfection/consistency”. This relatedness is indicated in the talk about development of the technological processes as well as the use of electronic test models.

### 3.3. Parents’ Qualification and Preparedness

The category “Parents’ qualification and preparedness” had two sub-categories, as described below.

#### 3.3.1. Preparing Students’ Parents for Online Education

According to the answers provided by the interviewees, the Ministry of Education was aware of the need to prepare students’ parents for online education. P4, from the NIET:

*We thought that parents would have the main role in encouraging their children’s attendance and engagement in the online lessons. We requested the school principals to take care of the parents’ issue through planning several face-to-face workshops, on the condition that they follow the safety directions of the ministry of education.*

Preparing students’ parents for online education is related to the dimension “administrative quality as transformation”. Students’ parents’ preparation for online education targets the success of the transformation of face-to-face education into distance education.

#### 3.3.2. Parents’ Interest in Developing Their Knowledge of Online Education

P3, from SQD, said:

*Some parents showed interest in knowing more about online learning, but others were reluctant to come to the school to develop their knowledge about online learning. We came to know that the children of parents who showed interest in online learning were more engaged in this learning than the children of parents who did not show this interest.*

P3′s description relates the development of parents’ knowledge of online education to the dimension “Administrative quality as perfection/consistency’’. She emphasized that the children of parents who were interested in knowing more about online learning were more consistent in their participation in online learning.

### 3.4. Follow-Up of Engagement and Attendance of Teachers and Students

The category “Follow up of engagement and attendance of teachers and students’ had two sub-categories that are described below.

#### 3.4.1. Following up the Attendance of Teachers

The respondents declared that the Ministry of Education was not only concerned with the teachers’, students’ and parents’ preparedness for distance teaching and learning, but it was also concerned with teachers’ and students’ attendance in online lessons. P7, from the AETIT, said:

*The attendance of teachers and students is followed up, and detailed reports are published. This has increased the opportunity to compare schools and directorates, in addition to asking directorates (school administrators) to request accountability from each teacher who has not been committed yet to the Teams.*

P7′s description of following up the attendance of teachers related this following up to the dimension “Administrative quality as exceptionality/excellence”. Thus, the Ministry of Education wanted to assess the excellence of teachers in online teaching, where this excellence was given also to schools regarding their overall distance educational activity.

#### 3.4.2. Following up the Engagement of Teachers

According to the answers provided by the interviewees, the Ministry of Education was not only concerned with the attendance of teachers and students in online learning, but also with their engagement in this learning. P9, from the AETIT, said: “The ministry of education followed up the engagement of teachers using the statistics in Teams. We thought that by publishing the statistics in the different governorates, teachers would be encouraged to engage more in online teaching”.

The above description also relates “Following up the engagement of teachers” to the previous dimension of excellence. Statistics were also used here to assess this excellence.

### 3.5. Enriching Online Content

The category “Enriching online content” had three sub-categories, as described below.

#### 3.5.1. Converting Learning Materials into Video Materials

On 10 March 2021, the official site of the Ministry of Education has called upon pedagogic supervisors, teachers, students and parents to convert learning materials into video materials and broadcast them through the Palestinian educational channel, in addition to adding them, periodically, to the Telegram channel, the Palestinian Education Gate and social media sites. On 17 May 2021, educational links for all classes appeared on the Ministry of Education’s site. These links included video-recorded lessons, video-based learning materials and educational packages.

“Converting learning materials into video materials” is related to the dimension “administrative quality as transformation”, as this conversion would support the transformation of the educational processes into distance ones.

#### 3.5.2. Putting Links to Educational Materials on School Sites

Collected data suggest that the Ministry of Education focused on putting links to educational materials on the schools’ sites. P9, from the AETIT, said:

*It was important for us to put links, on our site, to educational materials. To do so, we took advantage of the archived broadcasts on the Palestinian educational satellite in the Palestinian educational portal, for all classes. We expected these links to help teachers, students, and parents to return and watch them at any time, by easily clicking on the required lesson.*

“Putting links to educational materials on the school sites” is also related to the dimension “administrative quality as transformation”, as this provision of educational materials would support the transformation of the educational processes into distance ones.

#### 3.5.3. Recording the Synchronous Lessons and Uploading them for the Students and Parents

Teachers were requested to record their lessons and put them in the distance learning platform for their students to listen to when they need to do so. P2, a pedagogic supervisor said: *“Teachers were obliged to record their lesson through Teams and circulate the recordings to students and parents, which would help who had missed the synchronous lesson”.*

“Recording the synchronous lessons and uploading them for the students and parents” is related to the dimension “administrative quality as perfection/consistency”, where the perfection here includes responsiveness of the staff and their willingness to assist.

### 3.6. Enriching the Digital Curriculum

The category “Enriching the digital curriculum” had two sub-categories, as described below.

#### 3.6.1. Preparing Schedules for Online Lessons

School principals were requested to prepare schedules for online teaching. P2, from SQD, said:

*Several actions were undertaken by the principals in collaboration with the Directorate. Specific schedules for online lessons were planned for Basic Elementary school students (K4), for the fifth to tenth grades, and the eleventh to twelfth grades.*

“Preparing schedules for online lessons” is related to the dimension “administrative quality as perfection/consistency”, where preparing the schedules helps improve the occurrence of the lessons, as well as the preparation for these lessons.

#### 3.6.2. Providing Students with Interactive Books

The respondents declared that the Ministry of Education worked towards providing the students with interactive books. P1, from SQD, said: “The ministry of education worked hard to equip each student with an interactive book put on a CD”.

Providing students with interactive books is related to the administrative quality dimension “administrative quality as value for money”. This relatedness in indicated in P1′s use of the expression “worked hard to equip”, where working hard indicates, among other things, getting money that helps equip each student with an interactive book.

### 3.7. Strengthening Infrastructure

The category “Strengthening infrastructure” had four sub-categories, as described below.

#### 3.7.1. Increasing the Internet Speed in Schools

According to the answers provided by the interviewees, the Ministry of Education was concerned with the internet speed in schools. P5, from the NIET, said: “The ministry of education contacted the telecommunications company to increase the speed of the Internet in schools and universities”. P3, from SQD, said: “The principals were requested to buy routers to enable strong internet in the schools. The previous request was to ensure that teachers who do not have the internet at home would be able to work in the school”.

“Increasing the internet speed in schools’ is related to the dimension “administrative quality as value for money”. It seems that the budget of the Ministry of Education was constrained, so it negotiated the const of the increase in the internet speed.

#### 3.7.2. Providing the Schools with Laptops

Collected data suggest that the Ministry of Education took care of the schools’ infrastructure as well as the electronic devices for the students. P2, from SQD, said:

*Schools have been provided with computers through projects, where more than 50 laptops have been distributed to teachers and supervisors. An additional number will be provided shortly. 4500 tablets will be distributed soon, 2000 tablets will be distributed by UNICEF, 2000 from the Ministry of Social Development. Currently, we contact our partners to provide additional equipment.*

Here, too, “Providing the schools with laptops” is related to the dimension “administrative quality as value for money”. The money for the laptops was not provided by the Ministry of Education, but by its partners and by UNICEF.

#### 3.7.3. Fitting the Distance Learning Lesson to the Infrastructure

P6, from the NIET, said: “Instructions were issued to regulate the daily distance learning lessons. These instructions took into consideration the constrained equipment in the single-family”.

The above description is also related to the dimension “administrative quality as value for money”, where here, what is related is the daily distance learning lessons, i.e., the plan of these lessons and not the hardware, as mentioned before.

#### 3.7.4. Providing Teachers and Students with Emails

According to the answers provided by the interviewees, the Ministry of Education was also concerned with making sure that teachers and students have emails to enable effective communication between themselves and between each other. P8, from the AETIT, said:

*The department of Administration of Educational Technologies and Information Technology opened an official e-mail to all teachers and students. The Internet has been increased to 30 megabytes, 63% of schools, because of a technical problem with the Palestinian telecommunications company, 100% has not been reached, and 55% have been provided with Internet service, and the rest has not been completed due to delays in the bidding.*

“Providing teachers and students with emails” is related to the dimension “Administrative quality as exceptionality/excellence”, where the emails could support the excellence of the communication between all the participants in the distance education.

## 4. Discussion and Conclusions

Emergency education arose all of a sudden as a result of COVID-19, which constituted a challenge for schools ([41,42]), universities ([39,43]) and especially for the Ministries of Education, where the latter issue is the focus of the present research. The present research results indicate that the Palestinian Ministry of Education has played an important role in facing the Coronavirus pandemic and enabling education in this new context. Barham [24] argues that the positive impact of technology on students’ learning necessitates that the Palestinian MoEHE work to develop classroom environments that support the use of computer technology and its integration into learning. She further argues that “The Ministry’s ability to achieve this goal will be dependent on the availability of internal resources. A lack of resources in the country could be a challenge to the Ministry and may mean they will have to rely on outside donations to achieve their goal” (p. 20). During COVID-19 emergency education, the Palestinian MoEHE was aware of the need for appropriate resources for schools, teachers and learners. This made the Palestinian MoEHE try to provide resources that included hardware such as laptops and software such as electronic books.

During COVID-19 emergency education, the Palestinian MoEHE facilitated the educational processes needed for the success of students’ learning. Continuing this success would not be easy if the Palestinian MoEHE does not work very hard to maintain appropriate infrastructure and positive relationships between the Palestinian MoEHE itself, the local community and parents and the schools. Wahbeh [23] remarked that the schools’ administration points at the bureaucratic measures of the MoEHE as delaying the technology integration in schools. Hopefully, COVID-19 emergency education would have lessened these bureaucratic measures; otherwise, technology integration would stay limited in Palestinian schools. This act is in line with the Palestinian MoEHE’s view of ICT as an effective tool for shifting the teaching and learning processes from a teacher-centered approach to a student-centered approach ([24,26]).

An important means for successfully continuing technology integration in the Palestinian classroom as well as distance learning, especially blended learning, is the collaboration of the Palestinian MoEHE with the local community to be able to look after the needs of the schools regarding technology integration in the classroom. The role of the community in positively influencing technology use and distance education in schools is stressed in the previous literature. Manternach-Wigans [44] found that the local community could advance the use of technology in schools by providing resources such as money and technological devices. In addition, Byron and Gagliardi [45] say that “ICTs could facilitate the relationships between schools and the community and the process of transformation of schools in centers of sustainable development” (p. 43). This role of ICT indicates the importance of its utilization in schools as means for sustainable education in which schools and the community are in collaboration. We suggest that the Ministry of Education could be the main player in this collaboration, which will ensure the success of ICT integration in the schools, including distance education that schools need to adopt as one of their educational channels.

In addition to the above, working with the local community, the Palestinian MoEHE could overcome the moderate level of students’ perception of the utility of ICT for their learning. Through a questionnaire that had 12 items, Qaddumi, Bartram and Qashmar [46] studied students’ perceptions of the positive impact of ICT on their learning. They reported that four items had a very low level, one item had a low level, four items had a moderate level and three items had a high level. The previous results indicate the need to change Palestinian students’ perceptions about ICT’s role in their learning. This change could be achieved through collaboration with students’ parents, who need to take the main role in ICT integration in schools. This issue is also related to teachers’ engagement in positively influencing students’ perceptions of the utility of ICT for their learning. In addition, according to Almahasees et al. [47], adapting to online education has proven to be a challenge for students, including problems with technical and Internet communication, issues with data privacy and security. The previous studies indicate students’ difficulties in coping with distance learning during emergency education.

Qaddumi et al. [46] found that Palestinian teachers’ perceptions of the positive impact of technology on their teaching were high for most of the items. This high level indicates that teachers could contribute to the change of students’ perception of ICT towards a more positive one. It is recommended that parents and teachers collaborate, as this collaboration could benefit the educational processes in the classroom, even if this collaboration happens online [47]. The shared leadership of parents and teachers could result in a better taking of charge over students’ perceptions of the influence of technology on their learning, especially when parents and teachers have control over deciding on a method to positively change the students’ perceptions.

Another means for using ICT in education in Palestinian schools, especially distance education, is through collaboration with international organizations. During the COVID-19 pandemic, such organizations contributed to education in Palestine. The UN Office for the Coordination of Humanitarian Affairs (OCHA) [48] describes several funds donated to Palestine during 2018–2020, where part of them was intended for education. The Palestinian MoEHE could utilize some of these funds to improve the potentialities of the schools to integrate ICT, especially distance learning, in their classrooms.

Researchers point at the digital gap as one of the challenges most cited in the literature, with some students not having resources or the digital skills for online learning ([49,50]). Tomczyk and Walker [3] say that emergency e-learning highlighted class differences, where families with more resources were better able to provide their homes with suitable ICT equipment for effective e-learning. It seems that the Palestinian Ministry of Education was aware of the digital divide and the economic differences in Palestinian society, so it was concerned with equipping school students with laptops, expecting this move to influence positively students’ engagement in online learning.

The findings were enriched by the administrative quality framework [28]. It was shown that the processes performed by the Ministry of Education were according to this framework. Part of these processes had been performed for the sake of fitness for purpose, but another part was performed for the sake of perfection or excellence. The Ministry of Education had the “administrative quality as value for money” leading it the entire time, probably because of the economic constraints.

## 5. Limitations

The present research is limited by the small number of participants who agreed to be interviewed regarding the actions of Palestinian MoEHE to meet the challenges of COVID-19 emergency education. More in-depth research is needed to verify this issue; for example, the decisions made by the MoEHE and how they were affected by decisions taken in other countries. In addition, it is recommended that further research needs to study the actions of the Ministries of Education by using other theoretical frameworks specific to educational management.

Moreover, in-depth studies could utilize other data-collection methods such as focus groups and dilemma questionnaires. Quantitative studies could also contribute to the understanding of how Ministries of Education attempted to confront and make a smooth transition to distance education or/and emergency education. Moreover, special attention should be paid to the Ministries of Education bulletins for the education sector, for families and for communities. In the present research, we utilized some of the Palestinian Ministry of Education bulletins, but more scrutiny of these bulletins is needed. This would enable triangulation of the findings reported in the previous literature regarding the Ministry of Education’s efforts to confront emergency education in a specific country.

In addition to the above, the present research did not reveal much regarding the Ministry of Education’s efforts to smooth the move to distance learning in fields in which practical training cannot be replaced by distance learning. Future in-depth research is needed to study what Ministries of Education suggested in this case.

## Figures and Tables

**Table 1:** Background of the participants.

Participant	Department	Seniority at Work
P1	SQD	18 years
P2	SQD	15 years
P3	SQD	20 years
P4	NIET	25 years
P5	NIET	17 years
P6	NIET	21 years
P7	AETIT	13 years
P8	AETIT	21 years
P9	AETIT	23 years

**Table 2:** Analysis of collected data: categories, themes and examples.

Category	Themes	Terms Indicating the Theme
Students’ preparedness and attendance	Concern with students’ preparedness for distance learning	Training-students-distance
	Concern with Kindergarten to 2nd Grade (K2) students	K2-students
	Concern for students’ preparedness for special occasions	Students-preparedness-occasion
Teacher’s skills and qualification	Qualifying teachers for distance teaching	Qualify-teacher-distance
	Developing teachers’ skills in the use of technological tools	Teacher-skill-technology/tool
Parents’ qualification and preparedness:	Preparing students’ parents for online education	Prepare-parents-online
	Parents’ interest in developing their knowledge of online education	Parents-knowledge-online
Follow-up of engagement and attendance of teachers and students	Following up the attendance of teachers	Follow up-teacher-attendance
	Following up the engagement of teachers	Follow up-teacher-engagement
Enriching online content	Converting learning materials into video materials	Convert-learning material-video
	Putting links to educational materials on the school sites	Link-educational materials
	Recording the synchronous lessons and uploading them for the students and parents	Recoding-synchronous lectures
Enriching the digital curriculum	Preparing schedules for online lessons	Schedules-online
	Providing students with interactive books	Students-interactive books
Strengthening infrastructure	Increasing the internet speed in schools	Internet speed
	Providing the schools with laptops	Schools-laptops
	Fitting the distance learning lesson to the infrastructure	Fit-distance lessons-infrastructure
	Providing teachers and students with emails	Provide-teacher/student-email
